# A Computational-Experimental Approach Identifies Mutations That Enhance Surface Expression of an Oseltamivir-Resistant Influenza Neuraminidase

**DOI:** 10.1371/journal.pone.0022201

**Published:** 2011-07-20

**Authors:** Jesse D. Bloom, Jagannath S. Nayak, David Baltimore

**Affiliations:** Division of Biology, California Institute of Technology, Pasadena, California, United States of America; Erasmus Medical Center, Netherlands

## Abstract

The His274
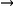
Tyr (H274Y) oseltamivir (Tamiflu) resistance mutation causes a substantial decrease in the total levels of surface-expressed neuraminidase protein and activity in early isolates of human seasonal H1N1 influenza, and in the swine-origin pandemic H1N1. In seasonal H1N1, H274Y only became widespread after the occurrence of secondary mutations that counteracted this decrease. H274Y is currently rare in pandemic H1N1, and it remains unclear whether secondary mutations exist that might similarly counteract the decreased neuraminidase surface expression associated with this resistance mutation in pandemic H1N1. Here we investigate the possibility of predicting such secondary mutations. We first test the ability of several computational approaches to retrospectively identify the secondary mutations that enhanced levels of surface-expressed neuraminidase protein and activity in seasonal H1N1 shortly before the emergence of oseltamivir resistance. We then use the most successful computational approach to predict a set of candidate secondary mutations to the pandemic H1N1 neuraminidase. We experimentally screen these mutations, and find that several of them do indeed partially counteract the decrease in neuraminidase surface expression caused by H274Y. Two of the secondary mutations together restore surface-expressed neuraminidase activity to wildtype levels, and also eliminate the very slight decrease in viral growth in tissue-culture caused by H274Y. Our work therefore demonstrates a combined computational-experimental approach for identifying mutations that enhance neuraminidase surface expression, and describes several specific mutations with the potential to be of relevance to the spread of oseltamivir resistance in pandemic H1N1.

## Introduction

In molecular evolution, multiple mutations are often required to confer an advantageous phenotypic change. Frequently, one mutation directly causes a beneficial functional alteration (such as a shift in substrate specificity or drug resistance), but is deleterious to protein-level properties such as folding, stability, or expression. A secondary mutation bolsters the protein-level properties damaged by the functional mutation, but by itself may confer no major adaptive benefit. Both mutations are needed to yield a protein that possesses the beneficial functional alteration and the requisite protein-level properties. Examples of this phenomenon may include the evolution of antibiotic resistance [1], [2], viral immune escape [3], steroid-receptor specificity [4], cytochrome P450 enzymatic activity [5], [6], HIV co-receptor usage [7], and influenza antiviral resistance [8].

When the functional mutation occurs first and is followed by a secondary mutation that repairs protein-level properties, the secondary mutation is typically referred to as “compensatory.” However, if an initial occurrence of a secondary mutation enables the protein to tolerate the subsequent functional mutation, the secondary mutation is referred to as “permissive” [4]. It is often impossible to determine which of these two scenarios actually occurred, but in some cases it appears that evolution proceeded via permissive mutations [4], [8]. This fact raises the tantalizing prospect that it may be possible to predict secondary mutations that could foreshadow future evolutionary change. In this paper, we explore the possibility of identifying mutations of possible relevance for the evolution of resistance to the neuraminidase-inhibitor oseltamivir (Tamiflu) in the 2009 swine-origin pandemic H1N1 influenza.

Resistance to oseltamivir is conferred on N1 influenza neuraminidases by the His274
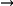
Tyr mutation (H274Y, N2 numbering), which causes a subtle structural change in the protein's active site that weakens the binding of oseltamivir [9]. Although H274Y could occasionally be identified in human seasonal H1N1 isolates from people taking oseltamivir [10], it was thought that this mutation was unlikely to spread appreciably. The reason for this view was that H274Y dramatically attenuated a variety of seasonal H1N1 strains in tissue culture and animal models, including A/WSN/1933 [11], A/Texas/36/1991 [12], A/New Caledonia/20/1999 [13], and A/Mississippi/3/2001 [14]. This attenuation coincided with a protein-level defect caused by H274Y that decreased the amount of neuraminidase expressed on the cell surface [8]. But by 2007, H274Y no longer detectably attenuated seasonal H1N1 isolates [14]–[16], and viruses carrying that mutation began to spread globally, going to near fixation in the 2008–2009 season [17]–[19]. This spread of resistance was preceded by secondary mutations that counteracted the decrease in neuraminidase surface expression caused by H274Y [8].

In the spring of 2009, human seasonal H1N1 was displaced by a new pandemic swine-origin H1N1 strain that continues to circulate globally [20], [21]. Currently, only about 1% of tested pandemic H1N1 isolates have carried H274Y [22], [23]. Most of these resistant isolates have been from immunocompromised patients or individuals taking oseltamivir, with only a few reported cases of H274Y virus being transmitted in healthy untreated adults [22], [23].

At the protein level, H274Y causes the same defect in neuraminidase surface expression observed in early seasonal H1N1. Specifically, H274Y causes a substantial decrease in the total protein and activity expressed on the surface of cells transfected with plasmids encoding pandemic H1N1 neuraminidase [8], while pandemic H1N1 viruses with H274Y possess between four and 10-fold less total neuraminidase activity [24]–[26]. However, as discussed immediately below, it remains unclear whether this decrease meaningfully attenuates viral fitness.

A number of experimental studies have compared the growth or transmission of matched isolates of wildtype and H274Y pandemic H1N1. In MDCK-derived cell lines, H274Y virus grew slightly but detectably worse than wildtype in five of eight cases [24], [27]–[30]; in the other three cases, there was no discernible difference [25], [29], [30]. H274Y virus grew slightly more poorly than its wildtype counterpart in differentiated human airway epithelium cells [25]. Upon direct inoculation of high doses into ferrets or mice, both wildtype and H274Y viruses replicated efficiently and caused disease in all studies [27]–[31]. Similarly, in all studies, both wildtype and H274Y viruses transmitted by direct contact with 100% efficiency between co-caged ferrets [29], [31] or guinea pigs [29]. Perhaps the most biologically relevant experimental measure of viral fitness is airborne transmission in ferrets or guinea pigs. In two of five comparisons, both wildtype and H274Y virus transmitted rapidly to all exposed animals in the experimental conditions used [29], [30]. But in the three comparisons without complete rapid transmission, the H274Y virus either transmitted markedly more slowly [30] or completely failed to infect some of the exposed animals [27], [29]. The authors of these studies differ about whether their results imply attenuation by H274Y – clearly, pandemic H1N1 is not severely crippled by the mutation as was early seasonal H1N1. This difference in the extent of attenuation caused by reduced neuraminidase levels could be due to as yet undefined differences elsewhere in the viral genome, such as in hemagglutinin receptor avidity [32]. However, from an evolutionary perspective, a reduction of viral fitness by even a few percent would likely prevent the spread of H274Y in pandemic H1N1, since only a small fraction of infected individuals use oseltamivir [33].

We therefore considered it worthwhile to investigate whether we could identify secondary mutations that counteract the decreased neuraminidase surface expression caused by H274Y in pandemic H1N1. We began by testing the ability of several computational approaches to retrospectively identify secondary mutations that increase the total surface-expressed neuraminidase activity in seasonal H1N1. We find that the PIPS computational approach [34] is the most capable of correctly identifying secondary mutations in this retrospective test. We then use this computational approach to predict 12 candidate secondary mutations to pandemic H1N1. We experimentally screen these mutants, and show that several of them do indeed increase the total surface-expressed protein and activity of H274Y pandemic H1N1 neuraminidase. Combining two of these secondary mutations with H274Y restores surface-expressed activity to approximately wildtype levels, and also rescues the modest attenutation that H274Y causes for viral growth in tissue culture. Our work therefore identifies several secondary mutations that have the potential to be of relevance for the evolution of oseltamivir resistance in pandemic H1N1.

## Results

### Retrospective testing of computational approaches for identifying important secondary mutations in seasonal H1N1

The goal of our study is to predict secondary mutations that enhance the surface-expressed activity and protein levels for H274Y pandemic H1N1 neuraminidase. There are various computational approaches that conceivably could be applied towards this goal. We therefore began by testing the ability of several computational approaches to retrospectively identify important secondary mutations from the evolution of seasonal H1N1 neuraminidase.

**Figure 1:**
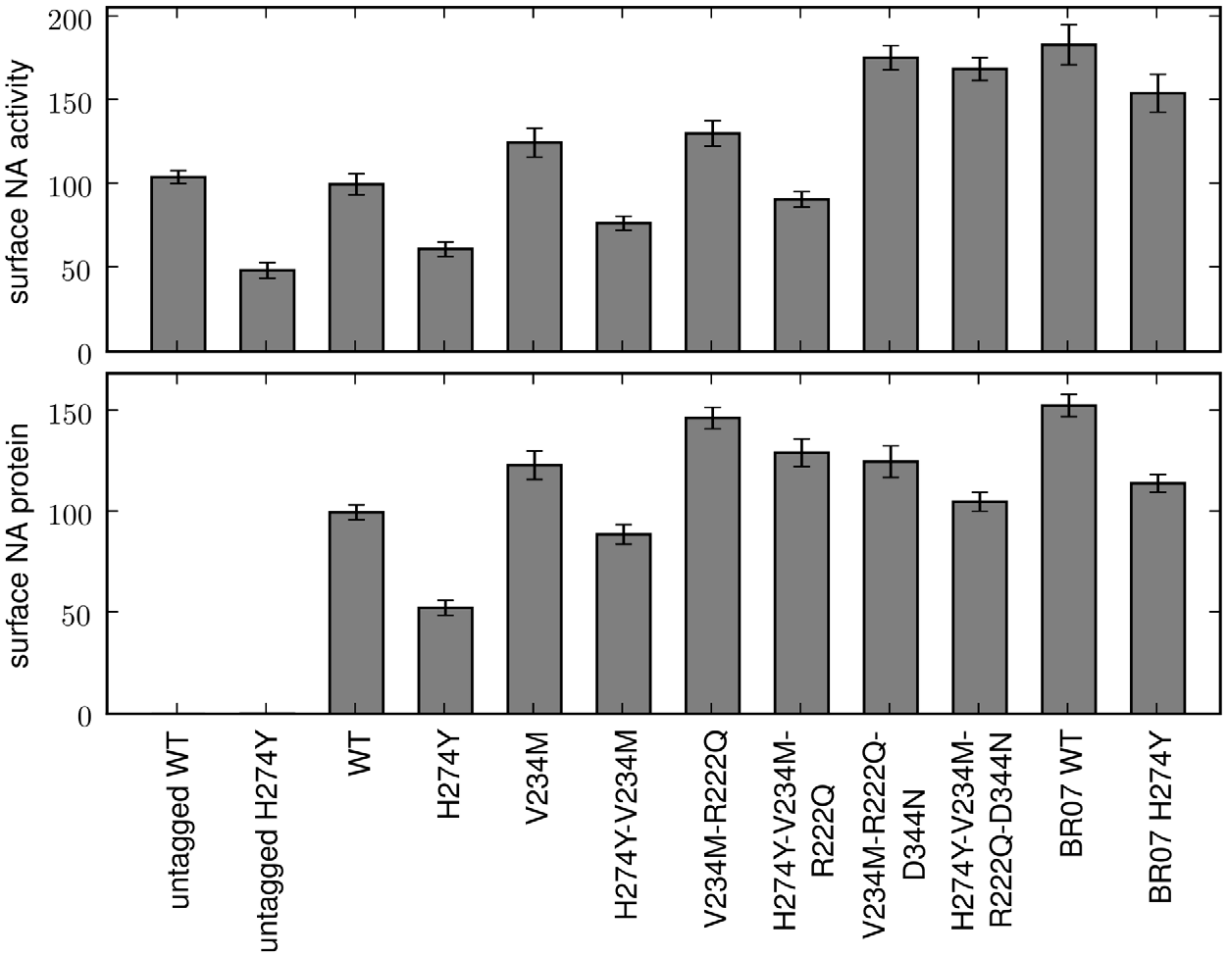
The three secondary mutations V234M, R222Q, and D344N largely explain the differences in total surface-expressed activity and protein between 1999 and 2007 seasonal H1N1 neuraminidases. Shown are wildtype (WT) and indicated mutants of the A/New Caledonia/20/1999 neuraminidase, in addition to WT and H274Y neuraminidases from the A/Brisbane/59/2007 (BR07) strain. All neuraminidases contain C-terminal epitope tags, except for the untagged WT and H274Y A/New Caledonia/20/1999 variants. For the measurements, 293T cells were transfected with plasmids encoding the neuraminidase proteins. After 20 hours, the cells were assayed for the total surface-expressed neuraminidase activity (top panel) or protein using an antibody against the epitope tag (bottom panel). Bars show the mean and standard error for at least six replicates.

The A/New Caledonia/20/1999 seasonal H1N1 strain is attenuated by H274Y [13], while the A/Brisbane/59/2007 strain is not attenuated by this mutation [14] and is an immediate ancestor of the lineage of oseltamivir-resistant viruses that went to fixation beginning in 2007. We performed assays to measure both the total surface-expressed neuraminidase activity and protein levels in mammalian cells transfected with plasmids encoding wildtype and H274Y neuraminidase proteins from these two strains. As described previously [8], H274Y caused an approximately two-fold decrease in surface-expressed neuraminidase protein and activity for the 1999 strain (Figure 1). In comparison, the wildtype 2007 neuraminidase was expressed on the cell surface at over 1.5-fold higher levels than its 1999 counterpart, and the relative magnitude of the decrease caused by H274Y was substantially smaller (Figure 1).

A total of 12 amino acid mutations separate the neuraminidases from these strains (H45N, V48I, K78E, E214G, R222Q, V234M, G249K, T287I, K329E, D344N, G354D, and D382N; N1 numbering). Two of these mutations (R222Q and V234M) have been shown experimentally to be sufficient to alleviate the attenuation of viral growth in tissue culture caused by H274Y in the background of the 1999 neuraminidase [8]. A third mutation (D344N) has been suggested to enhance neuraminidase substrate affinity [15], [35], [36]. We progressively added these mutations to the 1999 neuraminidase in the order that they appeared in natural sequences (V234M, then R222Q, then D344N). When all three mutations were added to the 1999 neuraminidase, it exhibited similar levels of total surface-expressed protein and activity to the 2007 neuraminidase, both in the presence and absence of H274Y (Figure 1). Of the remaining mutations, three (V48I, E214G, and D382N) have been tested previously [8]. In the background of an H274Y seasonal H1N1 neuraminidase, none of these mutations caused a substantial change in surface-expressed neuraminidase protein or activity. Since the divergence in surface-expressed protein and activity between 1999 and 2007 is explained by the three mutations R222Q, V234M, and D344N, for the purpose of the retrospective testing in this section, we placed these three mutations in one group. We then placed all of the remaining mutations in another group – although we stress that some of these remaining mutations have not been explicitly tested for their effect on neuraminidase surface-expressed activity.

We next sought to test whether computational approaches could identify the three known enhancing mutations from the complete set of mutations that separated the 1999 and 2007 strains. We reasoned that a computational approach that could correctly identify these three mutations might also be able to predict new mutations that enhance the surface expression of pandemic H1N1 neuraminidase. Because several of the candidate computational approaches utilize structural data, we restricted the analysis to the mutations that occurred in the crystallized [37] ectodomain of the neuraminidase (this excludes mutations H45N, V48I, and K78E). Our test therefore consisted of assessing the ability of the computational approaches to distinguish R222Q, V234M, and D344N from the remaining six ectodomain mutations (E214G, G249K, T287I, K329E, G354D, and D382N) that occurred during the divergence of the 1999 and 2007 strains.

We tested four different computational approaches. CUPSAT is a computer program that combines structural information with statistically derived potentials to predict the changes in protein stability associated with amino acid mutations [38]. FoldX is a computer program that uses a full atomic description of a protein's structure to predict mutational effects on protein stability [39]. The “consensus” approach assumes that the individual contribution of a mutation has a direct logarithmic (Boltzmann-like) relationship to its frequency in a sequence alignment of homologous proteins, such that the consensus residue is always assumed to be the most favorable [40]–[42]. Finally, PIPS is a method that we developed to infer mutational effects based on an analysis of protein phylogenies, and which has been shown to be able to predict secondary mutations that alleviate temperate-sensitive defects in influenza hemagglutinin [34]. The improved implementation of the PIPS approach used here is described in detail in the Materials and Methods section, as are the datasets used for the CUPSAT, FoldX, and consensus predictions.

**Figure 2:**
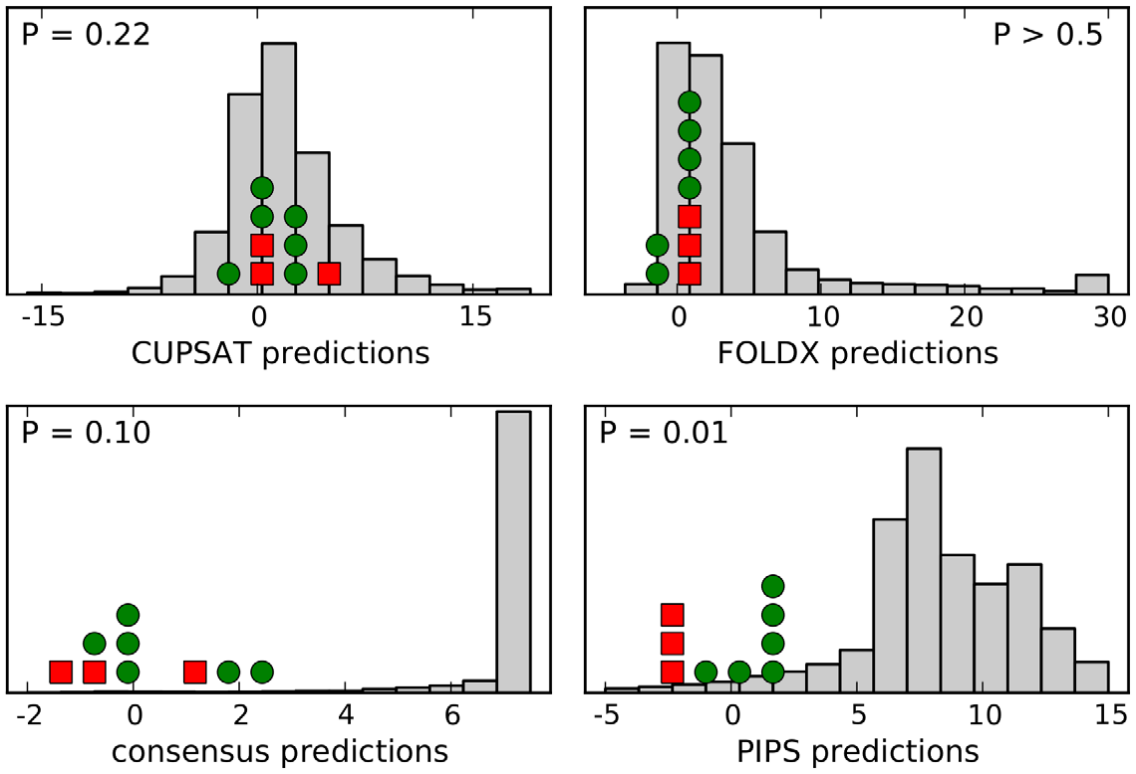
PIPS is the most effective computational approach for retrospectively identifying the secondary mutations that increased seasonal H1N1 neuraminidase surface expression and activity. The histograms show the distribution of predicted effects for all possible single amino-acid mutations to the A/New Caledonia/20/1999 neuraminidase, for each of the four computational approaches (CUPSAT, FOLDX, the consensus approach, and PIPS). The A/Brisbane/59/2007 strain contains nine mutations in the crystallized ectodomain portion of the neuraminidase relative to the A/New Caledonia/20/1999 strain. The three mutations that were experimentally show to enhance neuraminidase surface expression or activity (R222Q, V234M, and D344N) are indicated with red squares, while the other six mutations are indicated with green circles. The units for the different prediction methods are arbitrary, but in all cases more negative numbers correspond to mutations that are predicted to be more favorable. Shown are one-sided 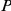-values for the hypothesis that the prediction method assigns more negative values to the known enhancing mutations (red squares) than the other six mutations (green circles), as determined using the Mann-Whitney test. The most successful computational approach appears to be PIPS, which correctly places all three red squares to the left of all six green circles.

Figure 2 shows the ability of each of the four computational approaches to distinguish R222Q, V234M, and D344N from the other six mutations. Neither CUPSAT nor FoldX showed any efficacy. Both of these methods placed the predicted effects of the nine actual ectodomain mutations near the center of the distribution for all possible neuraminidase mutations, and failed to separate R222Q, V234M, and D344N from the other six mutations. The consensus approach did identify the nine actual ectodomain mutations as being among the most preferable of all possible mutations, although this is a somewhat tautological result since by construction the approach prefers mutations that are prevalent in natural sequences. However, the consensus approach failed to separate R222Q, V234M, and D344N from the other six mutations. The PIPS approach was clearly the most successful. It classified the nine actual ectodomain mutations as being more preferable than most of the distribution of all possible mutations, and was further able to parse R222Q, V234M, and D344N as the most favorable of these nine mutations. We took this result as evidence that PIPS is the most promising approach for predicting mutations that enhance neuraminidase surface-expressed protein or activity.

### Prediction of mutations that counteract the neuraminidase defect associated with H274Y in pandemic H1N1

We next used the PIPS computational approach to predict the top 12 candidates for enhancing neuraminidase surface expression from the entire set of possible mutations to the ectodomain of the pandemic H1N1 A/California/4/2009 neuraminidase. These predictions are shown in Table 1. Plasmids were constructed encoding epitope-tagged H274Y neuraminidases with each of these secondary mutations. Among the secondary mutations discussed above as enhancing the surface-expressed activity of seasonal H1N1 neuraminidase, D344N is already present in the pandemic H1N1 neuraminidase. The identities of residues 222 and 234 in pandemic H1N1 are asparagine and valine, respectively. We therefore also constructed plasmids with the secondary mutations N222Q and V234M.

**Table 1:** Top twelve PIPS predicted neuraminidase mutations to pandemic H1N1.

mutation	PIPS prediction
N369K	−10.08
T289M	−7.79
V166A	−7.04
S366K	−6.74
P126N	−6.51
N386E	−6.45
V83M	−6.15
I389S	−6.01
G454N	−4.97
V106I	−4.95
R257K	−4.89
N221K	−4.87

Top predicted mutations to A/California/4/2009 neuraminidase, excluding mutations not in crystallized ectodomain and only considering the top prediction at each site. Mutations named in N1 numbering scheme.

**Figure 3:**
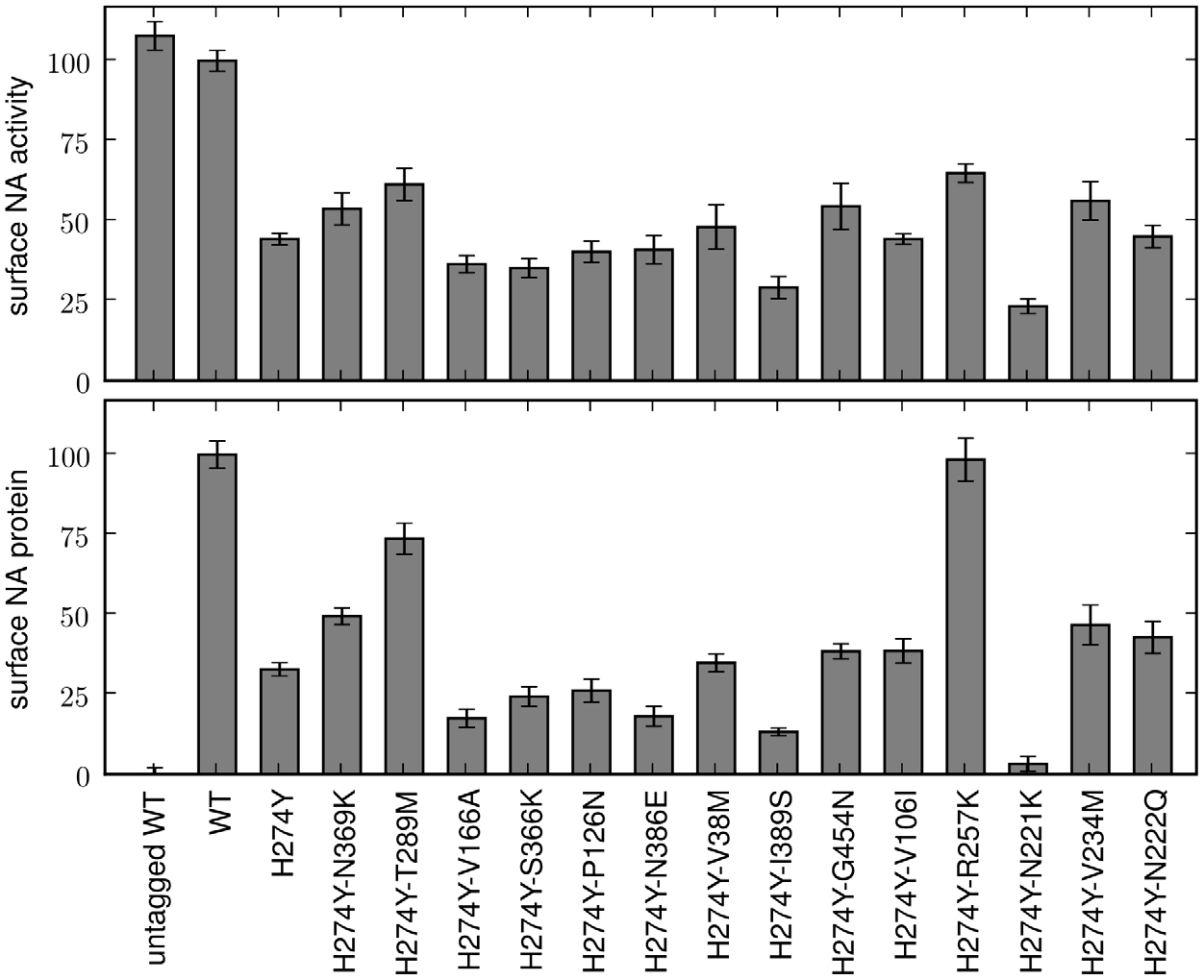
Several of the predicted secondary mutations partially counteract the decrease that H274Y causes in total surface-expressed activity and protein for the pandemic H1N1 neuraminidase. Shown are wildtype (WT) and indicated mutants of the A/California/4/2009 neuraminidase. All neuraminidases contain C-terminal epitope tags, except for the untagged WT. For the measurements, 293T cells were transfected with plasmids encoding the neuraminidase proteins. After 20 hours, the cells were assayed for the total surface-expressed neuraminidase activity (top panel) or protein using an antibody against the epitope tag (bottom panel). Bars show the mean and standard error for at least six replicates.

Each of these secondary mutations was tested for its effect on the total amount of neuraminidase activity and protein expressed on the surface of transfected cells (Figure 3). H274Y decreases surface-expressed activity and protein to less than half of wildtype levels. Several of the secondary mutations partially rescued this defect, with the strongest effects being mediated by R257K, T289M, N369K, and V234M (N1 numbering scheme). Other secondary mutations had no effect, or even decreased neuraminidase surface expression, indicating that the computational predictions are imperfect. Nonetheless, we considered it heartening that combining the computational predictions with a modest amount of experimental screening allowed us to identify several mutations of possible relevance.

**Figure 4:**
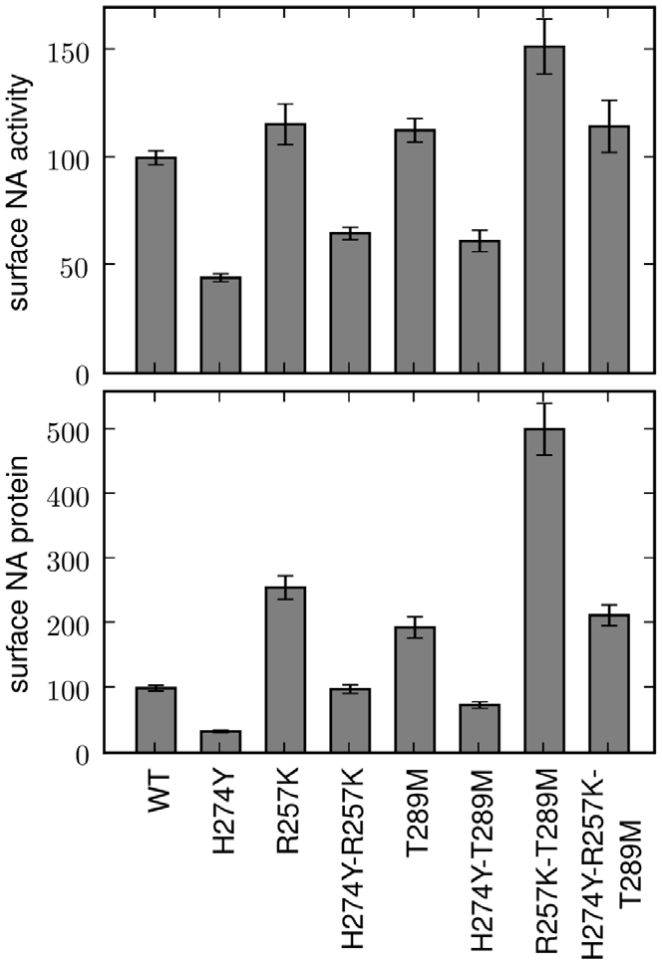
Combining several secondary mutations can fully counteract the effect of H274Y on surface-expressed pandemic H1N1 neuraminidase activity. Shown are wildtype (WT) and indicated mutants of the A/California/4/2009 neuraminidase, all containing C-terminal epitope tags. For the measurements, 293T cells were transfected with plasmids encoding the neuraminidase proteins. After 20 hours, the cells were assayed for the total surface-expressed neuraminidase activity (top panel) or protein using an antibody against the epitope tag (bottom panel). Bars show the mean and standard error for at least six replicates.

The two secondary mutations with the strongest effects were R257K and T289M. We constructed plasmids encoding both mutations in the background of either wildtype or H274Y, and measured the total surface-expressed neuraminidase activity and protein (Figure 4). Combining both R257K and T289M with H274Y rescued total surface-expressed activity to approximately wildtype levels. In the absence of H274Y, these two mutations increased total surface-expressed activity to levels 50% higher than wildtype. Interestingly, in both backgrounds, the effects of the R257K and T289M on the levels of surface-expressed protein were substantially larger than those on activity. The protein levels for the H274Y-R25K-T289M triple mutant were twice those of wildtype, while the levels for the double mutant without H274Y were five times higher than wildtype. This finding suggests that these secondary mutations either decrease the per-protein enzymatic activity, or cause a portion of the protein to reach the cell surface in an inactive form. However, this effect is outweighed by the overall increase in surface protein levels, such that the secondary mutations still enhance total surface-expressed activity.

### Secondary mutations eliminate the mild tissue-culture growth defect caused by H274Y in pandemic H1N1

To test the effects of the top candidate permissive mutations on viral growth, we used reverse genetics to generate pandemic H1N1 viruses carrying GFP in the PB1 segment [8]. These viruses derived their gene segments from the A/California/4/2009 strain, with the hemagglutinin containing the commonly occurring T197A mutation (which makes the sequence match that from the vaccine strain A/California/7/2009). We successfully rescued viruses with wildtype, H274Y, R257K-T289M, and H274Y-R257K-T289M neuraminidases.

**Figure 5:**
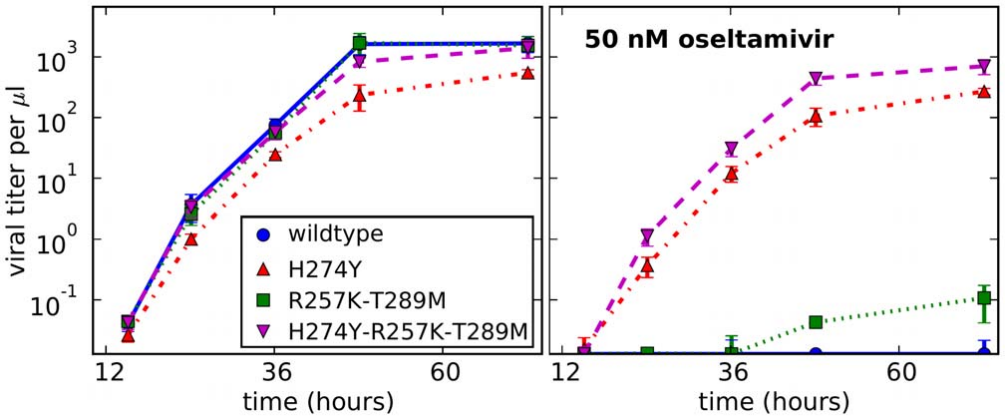
Growth in tissue-culture of pandemic H1N1 variants carrying neuraminidase mutations. The plot at left shows growth in media lacking oseltamivir, while the plot at right shows growth in media containing 50 nM oseltamivir. Viruses contain all genes from the A/California/4/2009 strain with the T197A mutation to hemagglutinin, with the exception of the PB1 segment which is engineered to carry GFP. MDCK-SIAT1-CMV-PB1 cells were infected with the viruses at initial multiplicities of infection of 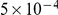 infectious particles per cell. At the indicated times, viral supernatants were harvested and titered on fresh cells. Shown are the mean and standard error for four replicates.

We performed viral growth assays in MDCK-SIAT1 cells that constitutively expressed the PB1 protein. As has been observed in the majority of previous studies [24], [27]–[30] with 2009 pandemic H1N1 strains, we found that H274Y caused a slight decrease in viral growth (Figure 5). Our results most closely resemble those obtained by [29] with the A/California/4/2009 strain, with the H274Y variant growing to slightly lower titers at all timepoints, with a maximal difference of about 10-fold.

However, the H274Y-R257K-T289M and R257K-T289M variants grew to titers similar to wildtype (Figure 5), suggesting that these two secondary mutations may rescue a slight attenuation in tissue-culture growth associated with H274Y. In the presence of 50 nM oseltamivir, neither the wildtype nor the R257K-T289M variants grew appreciably. But both the H274Y and H274Y-R257K-T289M variants grew as well as they had in the absence of oseltamivir. Therefore, the secondary mutations do not greatly affect viral resistance to oseltamivir *per se*, but may alleviate the slight tissue-culture growth defect caused by H274Y.

## Discussion

We have investigated the possibility of predicting secondary mutations that counteract the decreased neuraminidase surface expression associated with the H274Y oseltamivir resistance mutation in pandemic H1N1. We began with a retrospective test to find the most effective computational approach for identifying mutations that enhanced total surface-expressed activity and protein among all of neuraminidase mutations that occurred during the divergence of 1999 and 2007 strains of seasonal H1N1. We then used this computational approach to predict 12 new candidate mutations to pandemic H1N1. Three of these candidates (R257K, T289M, and N369K), as well as one of the secondary mutations from seasonal H1N1 (V234M), partially rescued the defect in surface-expressed neuraminidase activity and protein associated with H274Y in a 2009 pandemic H1N1 strain. Combining the two best candidates (R257K and T289M) with H274Y restored total surface-expressed activity to wildtype levels. These two mutations also appeared to rescue the slight defect in tissue-culture growth associated with H274Y in pandemic H1N1.

As discussed in the Introduction, the question of whether H274Y meaningfully attenuates pandemic H1N1 is a subject of continuing debate [24]–[31]. It therefore remains unclear whether the fact that H274Y pandemic H1N1 isolates have thus far been evolutionary dead ends [22], [23] is simply a matter of luck, or is because they are less fit than their oseltamivir-sensitive counterparts. Our results cannot resolve this question, which will ultimately be answered only by continuing to observe the natural evolution of the virus. However, our results do clearly demonstrate that a measurable phenotype associated with H274Y in pandemic H1N1 – a decrease in the total amount of surface-expressed neuraminidase protein and activity – has the potential to be counteracted by secondary mutations. Furthermore, we have identified four specific mutations (R257K, T289M, N369K, and V234M) with the potential to exert this effect. Note that this is unlikely to represent an exhaustive list of all mutations that enhance neuraminidase surface expression, since we only experimentally screened 14 of the nearly 9,000 possibilities. Nonetheless, these four mutations may be worthy of monitoring during surveillance of pandemic H1N1.

**Figure 6:**
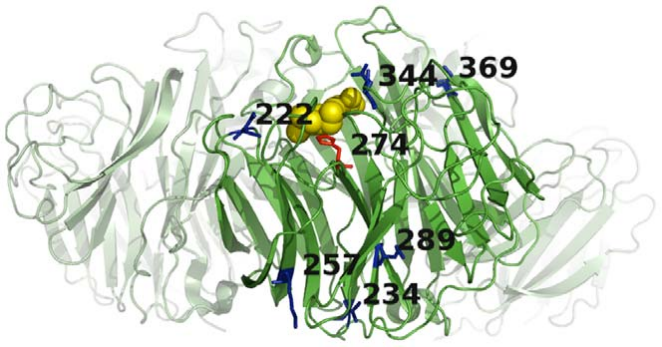
Sites of the mutations mapped onto the neuraminidases protein structure. Shown in dark green is one monomer from an N1 neuraminidase crystal structure ([37], PDB code 3BEQ]. Residue 274 (N2 numbering) is shown in red, and the sites of the secondary mutations (N1 numbering) are shown in blue. Oseltamivir (yellow spheres) is modeled in its binding site based on a related crystal structure ([83], PDB code 2HU0). The other three monomers of the full neuraminidase tetramer are shown in light green, based on modeling from a related crystal structure ([83], PDB code 2HU0). The image was rendered with PyMOL.

Regardless of the eventual fate of H274Y in pandemic H1N1, our findings are relevant to broader issues in protein evolution. We began this paper by describing the burgeoning set of examples where a mutation causes a beneficial phenotypic alteration only when it is paired with a secondary mutation. We further noted that these secondary mutations often act in a general manner by bolstering a protein-level property such as folding, stability, or expression, thereby alleviating defects caused by a variety of other mutations [1], [5], [43]–[48]. The potential for this phenomenon appears to be pervasive in influenza neuraminidase, as evidenced by the existence of multiple secondary mutations that partially counteract the decreased surface expression caused by H274Y. The exact biophysical mechanism remains unclear, and is an important area for further research. However, it is interesting to note that the mutations are scattered about the neuraminidase protein structure (Figure 6), and so appear to be generally promoting surface expression rather than forming a specific structural interaction with H274Y.

It is the generality of this “buffering” of protein properties that provides a basis for the strategy we used to identify potentially important secondary mutations. The PIPS computational approach is built on the idea that a single additive dimension captures the buffering effects of mutations on the whole set of evolutionarily constrained protein properties. Clearly this is a severe approximation, since mutations can have complex effects on each of these properties. But the approximation captures enough of the truth to be useful, since combining the resulting computational predictions with a modest amount of experimental screening was sufficient to identify secondary mutations that indeed enhanced neuraminidase surface expression. Whether any of these secondary mutations are actually found to play a role in increasing the permissiveness of pandemic H1N1 to oseltamivir resistance during future natural evolution will of course be the truest test of the practical value of this approach.

## Materials and Methods

### PIPS computational approach for predicting secondary mutations

The PIPS approach that we used to predict secondary neuraminidase mutations that might enhance neuraminidase surface expression is an improved version of that described in [34]. The approach is based on the idea that mutations frequently cause changes in protein-level properties that are under evolutionary constraint, such as stability, folding or expression. Previously [5], [34], [47], [49], we cast the evolutionarily relevant property solely as protein thermodynamic stability, 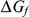. However, in the course of work by ourselves [8] and others [50], [51], it has become increasingly obvious that thermodynamic stability is not always the protein-level property under the strongest evolutionary constraint. We will therefore formalize a certain level of biophysical evasiveness by defining a variable 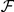, representing an approximate agglomeration of evolutionarily constrained properties such as thermodynamic stability, kinetic stability, folding efficiency, resistance to aggregation, intracellular trafficking, etc. In this formulation, 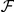 represents the best one-dimensional projection of all of these properties, to which in practice mutational effects are frequently [50], [52]–[56] but not always [45], [57] correlated. Describing each property individually would be more biophysically accurate, but would not be mathematically tractable in the approach that follows. The ultimate justification for a formalism based on the biophysically approximate variable 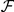 is experimental validation of some of the resulting predictions described here and in [34].

**Figure 7:**
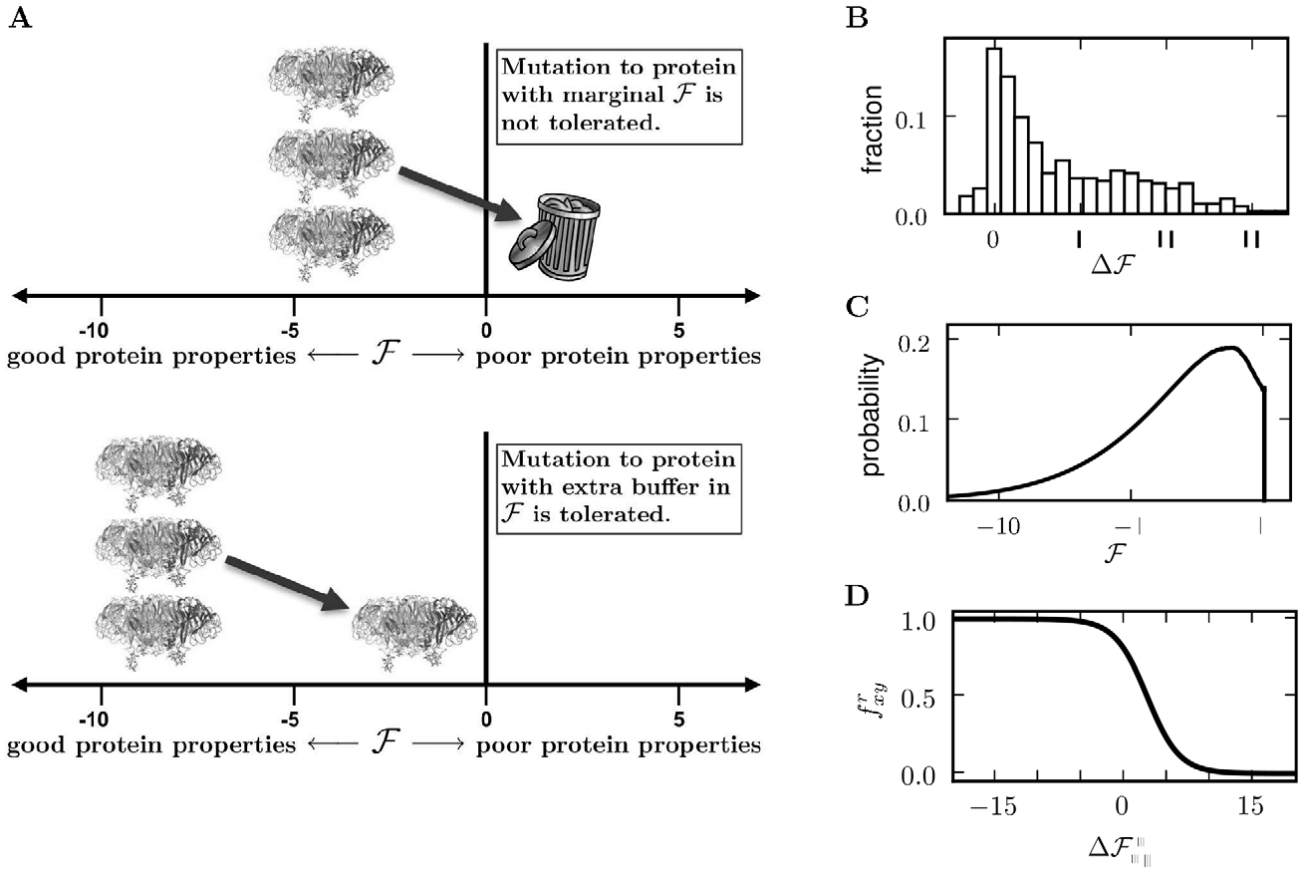
Rationale for assuming that the fixation probability of a mutation depends on its effect on evolutionarily constrained protein properties. (A) Evolution is assumed to select in a threshold manner for properties such as folding, stability, or expression (approximated by the variable 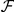). A mutation deleterious to 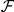 will not be tolerated by a protein that has a marginal value of 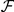 (top panel). But the same mutation is tolerated by a protein with an extra buffer in 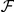 (bottom panel). (B) Most mutations are deleterious to 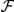, and therefore have positive 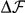 values. Shown is an example distribution of 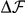 for all mutations to a protein, taken from [49]. (C) The time-averaged probability distribution of 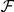 for an evolving protein will tend towards values just marginally below the threshold. Shown is an example of this distribution, taken from [49]. (D) As a consequence, mutations with negative 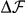 values will generally be tolerated, but those with positive 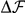 are less likely to be tolerated. Shown is a plot of the relationship between the probability 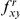 that mutating residue 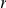 from 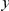 to 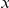 will be tolerated as a function of the associated 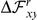 value, as defined in Equation 3.

More negative values of 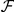 correspond to better protein properties, while more positive values correspond to worse properties. We assume that evolution selects to maintain 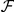 below some threshold (chosen here as zero) to ensure that the protein adopts and maintains its folded conformation. However, as long as 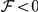, selection is indifferent to its exact value. When 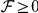, a protein is nonfunctional. Therefore, a mutation that worsens protein properties (increases 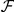) will not be tolerated by a protein that has a marginal value of 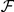 (top panel of Figure 7A). But the same mutation is tolerated by a protein with a larger margin in 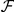 (bottom panel of Figure 7A). This relationship between 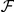 and mutational tolerance corresponds to the experimental observation that more stable proteins tend to be more robust to mutations [5], [47], [48], and the classic finding that certain mutations can “globally suppress” the deleterious effects of many other mutations by increasing stability or folding efficiency [43]–[46].

Each mutation is associated with a 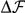 value, which is the difference between the 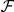 of the mutated protein and the wildtype one. Most mutations worsen protein properties, corresponding to an increase in 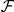, or a positive 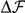 value. Figure 7B shows a representative distribution of 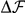 values for all mutations to a protein. The time-averaged probability distribution of 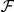 for an evolving protein is determined by the balance between the selection pressure to maintain 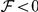 and the opposing pressure of mutations with mostly positive 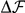 values. The exact distribution of 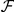 also depends on factors such as mutation rate, population size, and the specific 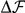 values associated with that protein [49], [58], [59]. However, the distribution will have the general feature that most of the time 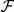 is just marginally below the selection threshold of zero. Figure 7C shows a representative time-averaged probability distribution of 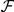.

The foregoing facts lead to an obvious relationship between a mutation's 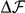 value and the probability that it will be fixed during neutral evolution. Specifically, let 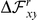 be the change in 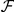 associated with mutating residue 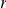 from 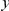 to 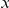. Given the above assumptions, when 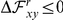, the mutation will always be selectively neutral, since it will never push 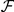 over the threshold of zero. On the other hand, when 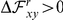, the mutation will only be selectively neutral if the protein possess a sufficient buffer in 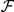, which will be the case when 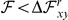. Given the time-averaged distribution of 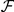 shown in Figure 7C, it is clear that mutations just slightly increasing 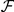 will frequently be neutral, while mutations with very large 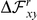 will only rarely be neutral. Let 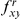 be the probability that the mutation is selectively neutral. The relationship between 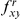 and 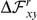 will have the general qualitative form shown in Figure 7D. We will use this relationship to infer 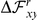 values from the mutational histories contained in protein phylogenies.

For each residue 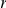, we want to infer the set 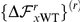 of the 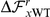 values for mutating the residue from its wildtype (WT) identity to some other residue 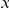. We will assume that the 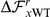 values for all residues are independent and additive, an assumption that although obviously imperfect is nonetheless likely to frequently be reasonable [60]–[65]. The specification of 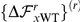 allows for calculation of arbitrary 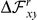 as
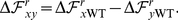
(1)The corresponding derivatives are
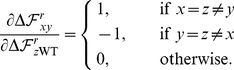
(2)


We have described 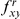 as the probability that the mutation of residue 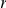 from 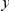 to 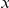 goes to fixation at the neutral expectation. Here we give an exact functional relationship between 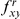 and 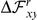. We have chosen this functional form arbitrarily, for simple reasons of mathematical convenience. However, it captures the key qualitative attributes discussed above. Specifically, we assume that
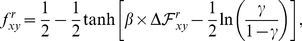
(3)where 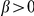 is a constant describing the steepness of the curve and 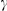 gives the value of 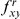 at 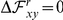. We use a range of 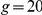 and constrain 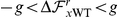. We set 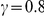, and then choose 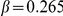 so that 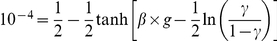. Equation 3 is plotted in Figure 7D. The corresponding derivatives are
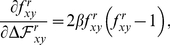
(4)and so by the chain rule,
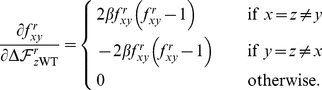
(5)


As in [34], define 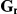 as the matrix with elements
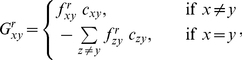
(6)where 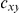 is the probability that a random nucleotide mutation to a codon for amino acid 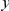 changes this codon to be for amino acid 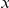. We refer to the set of all 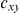 values as 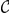. Again using the chain rule,
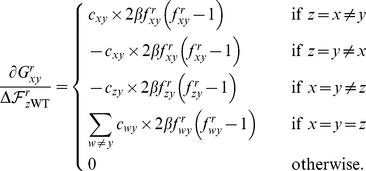
(7)


The probability that a substitution changes residue 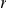 from 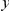 to 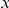 after an elapsed time 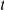 is given by element 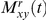 of the matrix 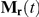 defined by
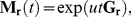
(8)where 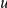 is the per codon mutation rate. Let 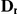 be the diagonal matrix with entries equal to the eigenvalues of 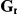, let 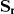 be the matrix with columns equal to the right eigenvectors of 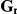, and let 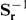 be the inverse of 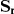, so that
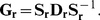
(9)The matrix 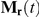 is conveniently computed as
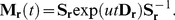
(10)The derivatives of 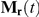 are given by [66] as
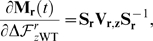
(11)where the elements of 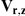 are
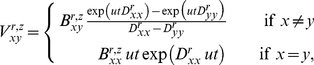
(12)where 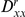 and 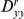 are the diagonal elements of 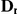 representing the eigenvalues of 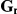, and 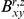 are the elements of the matrix 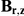 defined by
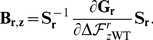
(13)


Let the probability 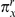 of finding residue 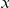 at position 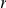 in the long-time limit be given by element 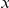 of the vector 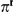. The vector 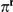 represents the stationary solution to Equation 8, and so is the probability vector (entries sum to one) that satisfies the eigenvector equation
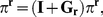
(14)where 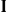 is the identity matrix. Given a value of 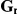, the uniqueness of 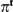 is guaranteed by the Perron-Frobenius theorems, since 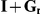 is a nonnegative and acyclic stochastic matrix. The derivatives of 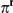 are given by [67] as
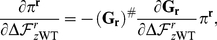
(15)where 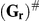 is the group inverse of 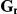 as described in [68].

In practice, we want to infer 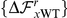 from a phylogeny built from a set of protein sequences. Let 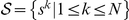 consists of 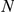 aligned homologous sequences of length 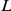, with 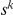 denoting the 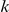
th sequence. For each sequence 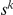, we know the identity 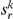 of the amino acid at position 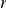 (where 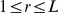). The set of amino acid identities for all 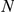 proteins at a single site 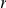 is denoted by 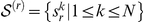. Let 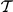 be the phylogenetic tree giving the relationship among these sequences. The probability of 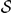 given 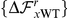, the set 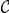 of 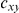 values, the mutation rate 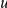, and the tree 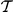, is the product of the per-site likelihoods,
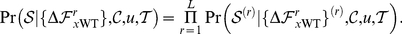
(16)


**Figure 8:**
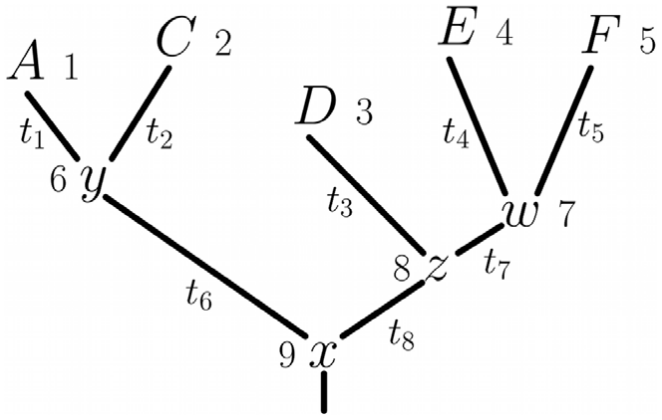
An example phylogenetic tree 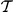. This tree shows the sequence data 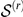 for five sequences at a single site 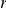. The amino acid codes at the tips of the branches (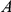, 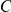, 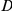, 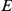, and 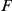) show the residue identities for the five sequences at this site. The variables at the internal nodes (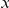, 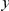, 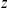, 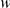) are the amino acid identities at the site for the ancestral sequences, and must be inferred. The numbers next to the nodes are unique identifiers for the nodes. The branch lengths (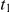, 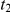,…) are proportional to the time since the divergence of the sequences.

For the example tree in Figure 8,
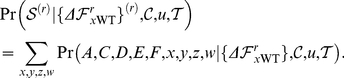
(17)Using the pruning approach of [69], [70],
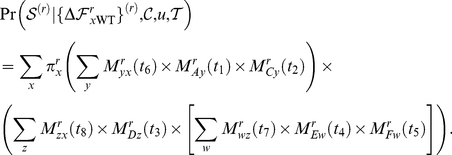
(18)


The derivatives of Equation 18 can be computed using the recursive nature of the likelihood calculation. This is most easily seen by introducing the notation where 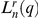 represents the likelihood that node 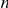 has residue 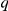 at position 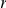 given all the data in the subtree rooted at node 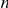. With this notation, Equation 18 is
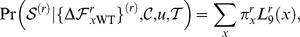
(19)where the likelihoods are calculated recursively down to the tree tips, so that for example,
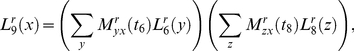
(20)and
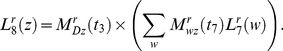
(21)Using this representation,
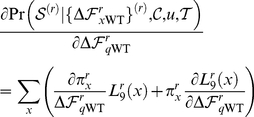
(22)where the derivatives of the 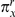 values are given by Equation 15, and the derivatives of the likelihoods are calculated recursively, as for example,
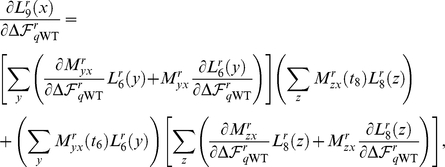
and
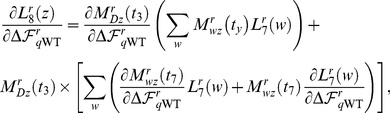
(24)where the derivatives of the 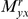 are given by Equation 11.

As discussed in [34], a prior probability distribution can be specified for each 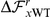 value. These priors can introduce specific biophysical knowledge as might be computed using molecular modeling programs, or can simply serve a “regularizing” role [71] to avoid overfitting the 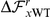 values. The priors also enforce the constraint that 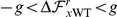. We define the prior probability distributions as beta distributions peaked at a prior estimate 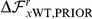 for the 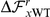 in question, and with the sum of the beta distribution 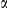 and 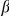 parameters equal to 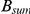,
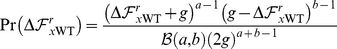
(25)where 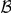 is the beta function, 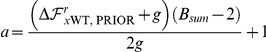, and 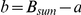. Note that 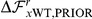 must satisfy 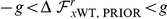. The derivative of Equation 25 is
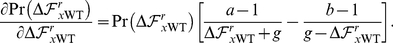
(26)The overall prior probability of the set of 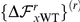 of 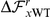 values for residue 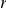 is simply the product of the prior probabilities for the individual 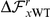 values,
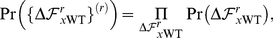
(27)so the derivative is
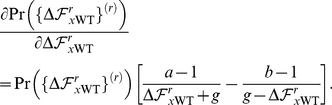
(28)


Equations 16 and 18 provide a method for computing 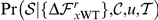. But goal is to infer the 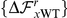, which is equivalent to computing 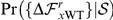. Using Bayes' Theorem,
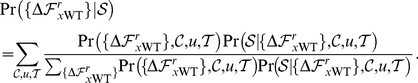
(29)Rather than solving for all of the unknown variables, here we will take the computational shortcut of using other methods to assign fixed values to 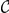, 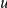, and 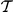, so that
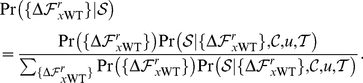
(30)Furthermore, rather than fully solving the right-hand side of Equation 30 as might in principle be done using Markov-chain Monte Carlo methods [72]–[74], we will simply compute the maximum *a posteriori* value 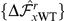 of 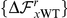, defined as
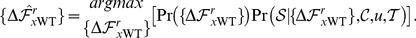
(31)Above we have provided equations for all of the derivatives necessary to perform this maximization using gradient-based techniques.

### Implementation of the computational approach in the PIPS program

A computer program that solves Equation 27 to infer 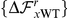 was written in the Python programming language and given the name PIPS (**P**hylogenetic **I**nference of **P**rotein **S**tability), version 1.0. This program and the raw data from the analyses described in this paper will be made freely available at http://labs.fhcrc.org/bloom/.

As input to the PIPS program, we used MUSCLE [75] to build a multiple-sequence alignment of all 3,731 unique full-length N1 neuraminidase protein sequences that were available for download from either NCBI's Influenza Virus Resource ([76], http://www.ncbi.nlm.nih.gov/genomes/FLU/FLU.html) or GISAID's EpiFlu Database ([77], http://platform.gisaid.org/) as of June 21, 2010. The aligned sequences were then used to build a neighbor-joining phylogenetic tree without a molecular clock, using the PHYLIP package [78]. This tree was used as input to the PIPS program.

The PIPS program was used to compute the 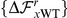 values for mutations to the neuraminidases from the seasonal H1N1 strain A/New Caledonia/20/1999 and the pandemic H1N1 strain A/California/4/2009. The prior probability distributions in Equation 22 were set so that all mutations had prior estimates of 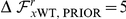, based on the idea that most mutations will be moderately deleterious to 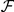. The value of 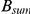 in Equation 22 was set to three. The mutation biases given by 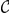 in Equation 6 were calculated by assuming that each amino acid is equally likely to be encoded by any of its possible codons, and that nucleotide mutations occur with a transition-to-transversion ratio of four. The value of 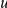 in Equation 8 was set to 10. The maximization in Equation 31 was performed using the conjugate-gradient algorithm. Although this algorithm is deterministic given specific starting values, there may be local maxima. Therefore, for each residue we performed five different maximizations starting from different randomly chosen 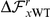 values, and used the values that gave the highest *a posteriori* probability as the final estimates.

Running the program in this fashion gave the PIPS predictions shown in Figure 2 for the specified mutations to the neuraminidase from A/New Caledonia/20/1999. For the mutations to the neuraminidase from A/California/4/2009, Table 1 lists the 12 mutations with the most negative predicted 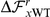 values, considering only the best mutation for each residue and only residues found in the ectodomain of the crystal structure of a closely related N1 neuraminidase ([37], PDB code 3BEQ).

### CUPSAT, FoldX, and consensus predictions

We also used CUPSAT, FoldX, and the consensus approach to predict the effects of mutations to the A/New Caledonia/20/1999 (H1N1) neuraminidase, as shown in Figure 2. Text files giving all of these predictions are available along with the PIPS program and raw data that are being made available at http://labs.fhcrc.org/bloom/.

CUPSAT and FoldX both take as their input a protein's structure. We used the crystal structure from PDB code 3BEQ [37], which is of the 1918 H1N1 influenza neuraminidase. This neuraminidase aligns to that of A/New Caledonia/20/1999 with no gaps and 89% protein identity over the 385 residues in the crystallized ectodomain. For the CUPSAT predictions, this protein structure was submitted to the webserver http://cupsat.tu-bs.de/cupsat/custompdb.htm to generate predictions for all single mutations. For FoldX, we made the predictions using the FoldX executable version 3.0 beta 4 for Mac OS X, as downloaded from http://foldx.crg.es/. The FoldX “RepairPDB” function was first run to refine the PDB structure. The predictions were then made using the default parameters and the “PositionScan” function. For the 89% of the residues in which the A/New Caledonia/20/1999 neuraminidase sequence exactly matched that in the 3BEQ crystal structure, the predicted mutational effects were simply the predictions for that mutation. For residues that differed between the two sequences, the predicted mutational effect was calculated as the predicted effect of mutating the PDB residue to the target amino acid minus the predicted effect of mutating the PDB residue to the A/New Caledonia/20/1999 residue. For both CUPSAT and FoldX, highly destabilizing mutations (values greater than the leftmost histogram bar shown in Figure 2) are counted in this last bar to avoid having to dramatically expand the x-axis of the plot in the positive direction.

For the consensus predictions, we used the same sequence data set of 3,731 full-length N1 neuraminidases that is described above for the PIPS program. The predicted effect of mutating a residue from amino acid 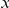 to 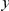 was calculated as 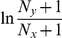 where 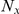 and 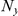 are the number of sequences that have amino acids 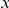 and 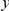 at that position, respectively. The one in the formula represents a single pseudocount added to each sequence tally to avoid undefined values for mutations to residues that are not present in the natural sequence alignment.

### Neuraminidase surface expression and activity assays

To test the effect of the predicted permissive mutations on the levels of surface-expressed neuraminidase activity and protein, we created plasmids encoding various mutants with C-terminal HA epitope tags. Each neuraminidase protein-coding sequence was directly fused to the epitope tag (YPYDVPDYA) and inserted into a plasmid (HDM) containing a CMV promoter and 5′ EcoRI/3′ NotI cloning sites, followed by an internal ribosome entry site (IRES) expressing the mCherry red fluorescent protein. As was previously observed [8], the addition of the C-terminal epitope tag led to at most a slight (less than 10%) decrease in the total surface-expressed neuraminidase activity relative to an untagged variant (Figures 1 and 3), indicating that the tag did not substantially alter the protein or activity levels. Plamids were constructed for all of the mutants of the A/New Caledonia/20/1999 neuraminidase shown in Figure 1 and all of the mutants of the A/California/4/2009 neuraminidase shown in Figures 3 and 4. In the naming of the mutations, H274Y was named in the N2 numbering scheme to adhere to historical convention – this is actually residue 275 in sequential numbering of the N1 neuraminidase. All of the other mutations are named according sequential N1 neuraminidase numbering.

For the assays, the plasmids were transfected into 293T cells in 12-well dishes that had been seeded at uniform densities of 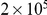 cells per well. At 20 hours post-transfection, the cells were collected using a very brief treatment with EDTA-trypsin, and resuspended in an isotonic assay buffer at pH 7.4, consisting of 15 mM MOPS, 145 mM sodium chloride, 2.7 mM potassium chloride, 4.0 mM calcium chloride, and 2% heat-inactivated fetal bovine serum. A fraction of these cells (5% of the total number collected per well) were then assayed for the total neuraminidase activity expressed on the cell surface using the fluorogenic MUNANA assay. For this assay, the cells were incubated with 0.1 mM MUNANA (Sigma M8639) in a total volume of 150 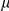
l in black 96-well plates at 37

C for 45 minutes. The reactions were quenched by adding 100 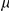
l of 150 mM sodium hydroxide in 84% ethanol. The fluorescence was read using a Tecan Safire 2 plate reader (excitation 360 nm, slit width 5 nm; emission 448 nm, slit width 20 nm). The activities were quantified as the fluorescence above the background from untransfected cells, normalized by the fraction of cells transfected with the plasmid as determined by flow cytometry for mCherry fluorescence as described below. Each bar for the activity measurements in Figures 1, 3, 4 represents the mean and standard error for at least six individual measurements.

A remaining fraction of the cells were stained with a fluorescently conjugated antibody against the epitope tag (Santa Cruz Biotechnology, HA probe F-7 Alexa-Fluor 647 conjugate, sc-7392 AF647, 1∶100 dilution). The stained cells were analyzed by flow cytometry to determine the fraction of cells expressing the mCherry protein (these are the cells transfected with the plasmid), and the mean signal from the antibody staining among these mCherry positive cells. The staining signal above background was assumed to be proportional to the amount of neuraminidase protein on the cell surface. Each bar for the stain measurements in Figures 1, 3, 4 represents the mean and standard error of at least six individual measurements.

### Viral growth assays

Reverse genetics plasmids for the A/California/4/2009 H1N1 strain were constructed by using reverse-transcriptase PCR to amplify the genome segments from total RNA extracted from virus obtained from the Biodefense and Emerging Infections Resource Repository (BEI Resources, catalog number NR-13658). The hemagglutinin gene for A/California/4/2009 was modified by adding the T197A mutation, since this mutation is present in the majority of 2009 pandemic H1N1 isolates including the A/California/7/2009 vaccine strain, and has been reported to aid in virus rescue by reverse genetics [79]. The gene segments were cloned into the BsmBI sites of the bidirectional RNA polymerase I/polymerase II cassette plasmid pHW2000 [80], which was kindly provided by Robert Webster of St. Jude Children's Research Hospital. Mutations to the neuraminidase were introduced by site-directed mutagenesis.

Virions carrying GFP in the PB1 segment were rescued as described in [8]. Briefly, the plasmid pHH-PB1flank-eGFP encodes a viral RNA with the untranslated regions and 80 terminal coding nucleotides from each end of the PB1 gene segment from A/WSN/33 influenza, with potential start codons mutated. This plasmid and the reverse genetics plasmids for the other seven influenza segments (PB2, PA, HA, NP, NA, M, and NS) were co-transfected into a co-culture of 293T (ATCC CRL11268) and MDCK-SIAT1 ([81], HPA Cultures 05071502) cells that constitutively expressed the A/WSN/33 PB1 protein under a CMV promoter (293T-CMV-PB1 and MDCK-SIAT1-CMV-PB1 cells), with the PB1-F2 peptide eliminated by introduction of a stop codon in the manner described by [82]. At 12 hours post-transfection, the cells were washed once with PBS and the media changed to influenza growth media (Opti-MEM I supplemented with 0.3% bovine serum albumin, 0.01% heat-inactivated fetal bovine serum, 100 U/ml penicillin, 100 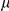
g/ml streptomycin, and 100 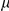
g/ml calcium chloride) containing 3 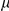
g/ml TPCK-treated trypsin. After another 60 hours, at which point essentially all cells had turned green and were undergoing visible cytopathic effect, the viruses were harvested by filtration through a 0.45 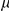
m filter. The viruses were titered by infecting MDCK-SIAT1-CMV-PB1 cells in influenza growth media, and then quantifying the percentage of GFP positive cells at 15 hours post-infection using flow cytometry. Each virus variant (wildtype, H274Y, R257K-T289M, and H274Y-R257K-T289M neuraminidase) was rescued and titered in duplicate.

For the growth assays, MDCK-SIAT1-CMV-PB1 cells were seeded in 6-well dishes so that they were at 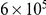 cells per well at the time of viral infection. Immediately before infection, the medium was changed to 3 ml of influenza growth media plus 3 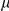
g/ml TPCK-trypsin. Some wells also contained 50 nM oseltamivir carboxylate (kindly provided by J. Smith and A. Perrin of F. Hoffmann-La Roche), as indicated in Figure 5. Each well was then infected with an amount of virus equal to 300 infectious particles according to the flow cytometry titering. At the time points indicated in the figures, supernatant was collected and the viral titer determined by flow cytometry titering on fresh MDCK-SIAT1-CMV-PB1 cells. Each point in the figures shows the mean and standard deviation for four total replicates, with two replicates performed with each of the two separate virus rescues. The exceptions are the measurements for the wildtype and R257K-T289M viruses in 50 nM oseltamivir, where only two total replicates were performed (one with each of the two separate virus rescues).
